# Correction of axial and rotational alignment after medial and lateral releases during balanced gap TKA: A clinical study of 54 patients

**DOI:** 10.3109/17453674.2010.483992

**Published:** 2010-05-21

**Authors:** Petra J C Heesterbeek, Ate B Wymenga

**Affiliations:** ^1^Department of Research, Development and Education; ^2^Department of Orthopaedics, Sint Maartenskliniek, Nijmegenthe Netherlands

## Abstract

**Background and purpose:**

Restoration of mechanical alignment after total knee arthroplasty can be achieved with ligament releases. Several previously described sequences and results achieved with cadaver knees, with measured resection implantation techniques, may not be applied to the balanced gap technique. We investigated the peroperative effect of stepwise soft tissue releases following the “tightest structure first” on leg axis in extension and femur rotation in flexion.

**Methods:**

During PCL-retaining total knee arthroplasty (TKA), using a balanced gap technique in 54 patients we determined the effect of each ligament release using a navigation system while the knee was distracted with a tensor in extension and flexion. The effect on alignment in extension and on femoral rotation in flexion was measured for each release separately.

**Results:**

In more than half of the patients, one or more ligament releases were necessary. Release of the posteromedial condyle led to a minor effect on leg axis in extension and femoral rotation in flexion, release of the superficial medial collateral ligament to a few degrees, mainly in extension. Release of the iliotibial tract led to a small correction of leg alignment in extension. There was no statistically significant difference in the alignment-correcting effect of a release dependent upon the sequence in which the structure was released.

**Interpretation:**

In PCL-retaining TKA, a stepwise “tightest structure first” protocol for ligament releases in extension with the balanced gap technique results in effective, gradual, alignment correction in extension, and limited femoral rotating effects in flexion.

## Introduction

Restoration of mechanical alignment after total knee arthroplasty (TKA) is necessary: postoperative imbalance of collateral ligaments and incorrect mechanical alignment can lead to early loosening of the prosthesis (Insall et al. 1985, Fehring et al. 2001). Correction of mechanical alignment can be achieved by soft tissue releases, and by the proximal tibial and distal femoral cuts. There are 2 main philosophies: a measured resection technique and a balanced gap technique. These techniques differ in procedure and rotational alignment of the femoral component. With measured resection techniques, the posterior and anterior bone cuts of the femur are performed at fixed, “measured”, angles referenced from anatomical landmarks of the femur. The 3 most commonly used measured resection techniques for the posterior and anterior femoral cuts use the transepicondylar axis, the anteroposterior line or Whiteside’s line, and the posterior condyles as reference. Ligament releases in extension are usually performed after implanting the prosthesis (Berger et al. 1993, Whiteside and Arima 1995, Poilvache et al. 1996, Olcott and Scott 2000, Katz et al. 2001). With a balanced gap technique, this sequence is reversed: after the proximal tibia cut the tight ligamentous structures are released first, one-by-one, tightest first, to align the leg in extension. Then a lamina spreader or a tensor can be used to tension the ligaments and the anterior and posterior femoral bone cuts are made parallel to the tibia cut based on the ligament tension. No further releases are performed in flexion (Insall et al. 1976, 1985). As a consequence of this balanced gap technique (and in contrast to a measured resection technique), the rotation of the femoral component can vary freely within the restrictions of the soft tissue structures.

From laboratory studies, we have learned that releases of soft tissue structures in extension also have a considerable or an even more pronounced effect on the flexion gap (Matsueda et al. 1999, Krackow and Mihalko 1999a,b, Saeki et al. 2001, Kanamiya et al. 2002, Mihalko et al. 2003, Luring et al. 2005, 2006a,b). Also, clinical studies investigating the effect of medial and/or lateral ligament releases on the extension and flexion gap have confirmed effects in both extension and flexion (Whiteside et al. 2000, Yagishita et al. 2003, Mihalko et al. 2003, Clarke et al. 2005). However, all the TKAs in these studies were performed using posterior-stabilized (PS) prostheses with a measured resection technique. Because the balanced gap technique with a PCL-retaining implant is fundamentally different to the measured resection technique with a PS implant, the sequences and results described may not be applied to the balanced gap technique, in which we were interested. Furthermore, we were interested in the effects of releases on femoral rotation since ligament releases in extension together with the “free” femoral component rotation of the balanced gap technique could result in an internally rotated femoral component (Figure 1) with possible patellofemoral maltracking (Fehring 2000).

**Figure 1.**
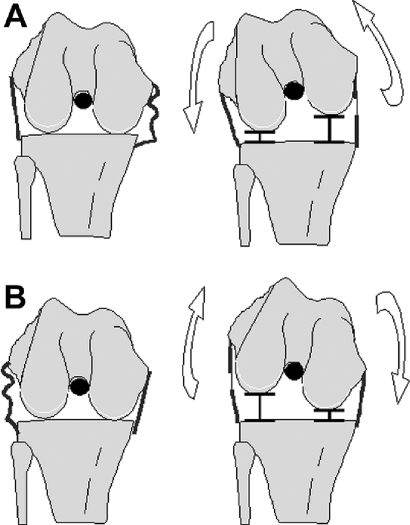
A. After a medial release (already released in the drawing to the left), when a tensor is inserted in the knee the femur would theoretically exorotate as a result of the loosened medial ligament structure. B. After a lateral release (already released in the drawing to the left), when a tensor is inserted in the knee the femur would theoretically endorotate as a result of the loosened lateral ligament structure.

Since no clinical studies were available on the effects of releases with PCL-retaining TKA using a balanced gap technique on alignment in extension and femoral rotation in flexion, we decided to investigate these effects in a clinical study. Thus, we investigated the effects of stepwise soft tissue releases according to the “tightest structure first” sequence on leg axis in extension and on femur endo/exorotation in flexion during PCL-retaining TKA with a balanced gap technique.

## Patients and methods

### Patients

In this institutional review board-approved study, 54 patients with osteoarthritis of the knee gave informed consent to participate. All patients received the same fixed-bearing, PCL-retaining total knee prosthesis (balanSys; Mathys AG, Bettlach, Switzerland), and surgeries were performed by one surgeon (AW). Inclusion criteria were: primary osteoarthritis, indication for cruciate-retaining TKA, and fixed deformity of less than 10°. Exclusion criteria were: traumatic osteoarthritis, BMI of > 30, and ipsilateral total hip replacement. For all patients, a preoperative standing, long leg radiograph was obtained.

The local ethics committee approved the study on October 18, 2004. It is registered as CMO-nr: 2004/180 at the Commissie Mensgebonden Onderzoek Regio Arnhem-Nijmegen.

### Navigation system

All surgeries were navigated using a CT-free navigation system (Surgetics; Praxim, La Tronche, France). This system is based on an optical tracking unit that detects markers on reference frames attached to the bone and instruments with an infrared camera system. Alignment of the leg (hip-knee-ankle axis) can be determined in real time in extension and in 90° of flexion (also referred to as femoral rotation). Movements of 0.5 mm or 0.5° could be detected by the camera, and the total accuracy of the system was 1 mm or 1° (information from the manufacturer).

### Surgical technique and measurements

We followed the standard surgical protocol for navigated surgery. The surgical approach was either medial or lateral, depending on whether the knee had a varus or valgus alignment. The bone was prepared and peripheral osteophytes were removed. The tibia cut was made with preservation of the PCL insertion by a bony island through a so-called “V-cut”. The tibial slope was set at 7°. After the tibia cut, the leg was placed in extension and a bi-compartmental tensor (balanSys) (Figure 2) was inserted into the knee at 150 N, with an everted patella. The force was applied perpendicular to the blades of the tensor and perpendicular to the tibial slope. The mechanical leg axis was displayed by the navigation system and recorded. The leg axis in extension was defined as the medial angle between the mechanical femur and tibia axes. The mechanical femur axis is the line between the center of rotation of the hip and the femoral notch. The tibia axis is determined as the line between the tibial spine (before the tibia cut) and the center of the ankle that is calculated using the digitized medial and lateral malleoli. An angle of < 180° was defined as varus alignment, and > 180° as valgus alignment. Subsequently, the knee was flexed to 90° and the tensor was inserted at 100 N. Again, the leg axis was recorded—this being the rotation of the femur in flexion. Rotation of the femur in flexion was determined using the surgical transepicondylar axis that goes through the medial and lateral epicondyles digitized by the surgeon during the standard surgical work flow.

**Figure 2.**
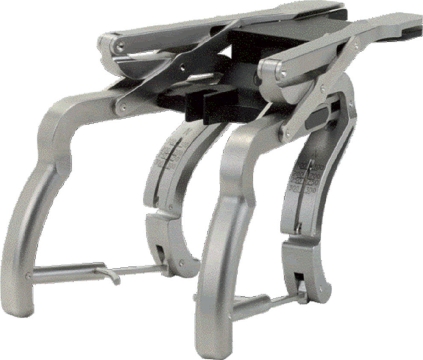
Double-bladed calibrated tensor (balanSys; Mathys AG, Bettlach, Switzerland). A quantified amount of vertically-oriented force can be applied independently to the medial and lateral compartments.

If the leg axis in extension was 180°, no ligament release was performed and the surgeon continued the surgical protocol. If the leg axis in extension was in varus alignment or in valgus alignment, the surgeon extended the knee and inserted the tensor set at 150 N. The tightest structure was identified and released (without the tensor in place). All medial structures were released from the tibia as well as the iliotibial tract (IT) and the posterolateral corner (PLC); the popliteus tendon (POP) was released from the femoral insertion. After this first release, the tensor was inserted into the knee in extension (150 N) and after that in flexion (100 N), and again the leg axis in extension and femoral rotation in flexion were recorded. This procedure was repeated as necessary for a second or third ligament structure until the leg axis in extension was 180°. For multiple ligament releases, the sequence of the released structures was recorded. No releases were performed in flexion.

### Data analysis and statistics

Analyses were carried out to determine the effect of each release step on the leg axis in both extension and flexion. Box plots were used to compare the leg axis in extension and femur rotation in flexion between each release step. The height of the box is the interquartile range and represents 50% of the values. The median is the horizontal line across each box. The vertical lines represent the minimum and maximum values. The STATA software package version 10.1 was used to create the box plots. For differences in preoperative leg alignment on standing, long leg radiographs for each release category were tested using the Kruskal-Wallis test. Differences between the effects produced by releases were tested with the Mann-Whitney test to determine whether the sequence (first or second) influenced the effect; p-values of ≤ 0.05 were considered statistically significant.

## Results

54 patients (35 females) underwent surgery. In 23 patients, leg axis in extension was 180° and no ligament release was necessary to align the leg; this group is referred to as “no releases”. In 31 patients, the leg axis in extension was not 180° and these patients needed 1 or more ligament releases. All medial ligament releases were performed in varus knees and all lateral ligament releases were performed in valgus knees. The median leg alignment on the preoperative standing radiograph was significantly different for the knees in the 3 release categories (p < 0.001). Median preoperative leg alignment on the preoperative standing radiograph was 3° valgus (range 11° valgus to 13° varus) for “no releases”, 7° varus (range 0° to 14° varus) for “medial releases”, and 4.5° valgus (range 1° valgus to 8° valgus) for “lateral releases” (Table). After all releases had been performed, all patients had a correct leg axis in extension of 180°, as measured with the navigation system.

**Table:** Patient characteristics and median alignment in extension and median femoral rotation in flexion before and after all releases, for the 3 release categories

	No releases (n = 23)	Medial ligament release (n = 24)	Lateral ligament release (n = 7)
Sex (M/F)	6/17	13/11	0/7
Side (L/R)	10/13	11/13	3/4
Median age at surgery (range)	67 (44–79)	60 (47–84)	62 (54–81)
Median alignment in extension			
pre-release (range)	180 (179–182)	177 (173–179)	182 (182–184)
after all releases (range)	N/A	180 (178–181)	180 (179–182)
Median femoral rotation in flexion			
pre-release (range)	3.8 (0.5–10.2)	4.5 (-0.7–8.1)	4.5 (3.1–13.0)
after all releases (range)	N/A	2.4 (-4.4–7.9)	4.7 (3.7–14.6)
Median amount of releases (range)	0	2 (1–3)	1 (1–2)

### Medial ligament releases (n = 24)

The posteromedial corner (PMC) was the most frequently released structure (21 times), followed by the superficial medial collateral ligament (SMCL) (16 times). The semimembranosus (SEM) was the least frequently released structure (2 times). 11 patients had 1 ligament release, 11 patients had 2 ligament releases, and 2 patients had 3.

When the PMC was released as first structure (n = 15), this led to a median change of 1° in alignment in extension (Figure 3). Median subsequent change in femoral rotation in flexion after this release was 0.3° exorotation (Figure 4). After this first structure, release of the SMCL was necessary in 9 of 15 patients. The median additional change in leg axis after SMCL release in extension was 3°; the median additional change in femoral rotation in flexion was 2.1° exorotation.

**Figure 3.**
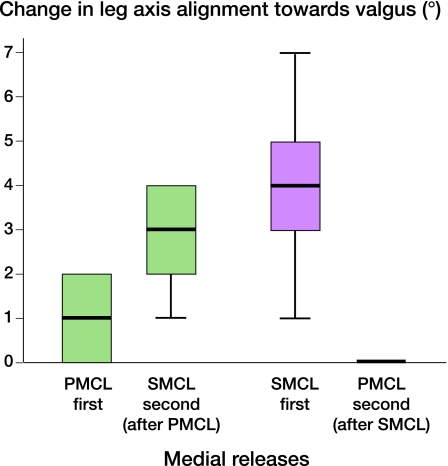
Box plot of change in leg axis after PMC release (n = 15) and subsequent SMCL release (n = 9) (green boxes, on the left), and after SMCL release (n = 4) and subsequent PMC release (n = 1) (pink boxes, on the right). The median is shown as a horizontal line across each box. The vertical lines represent the minimum and maximum values.

**Figure 4.**
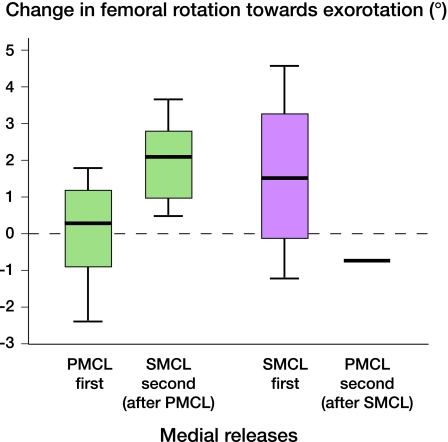
Box plot of change in femoral rotation after PMC release (n = 15) and subsequent SMCL release (n = 9) (green boxes, on the left), and after SMCL release (n = 4) and subsequent PMC release (n = 1) (pink boxes, on the right). The median is shown as a horizontal line across each box. The vertical lines represent the minimum and maximum values.

In 4 patients, the first released structure was the SMCL. The release of this structure led to a 4° median change in alignment in extension; the median change in femoral rotation in flexion was 1.5° exorotation. After this primary release, the PMC was subsequently released in 1 patient, leading to no additional change in leg axis and an additional change of 0.7° endorotation of the femur in flexion.

There were no statistically significant differences in change in alignment depending upon whether a ligament was released as first or as second structure (p = 0.5 and p = 0.7 for change in alignment in extension and rotation in flexion, respectively, after SMCL release and p = 0.4 and p = 0.6 for change in alignment in extension and rotation in flexion after PMC release).

### Lateral ligament releases (n = 7)

4 valgus knees required 1 ligament release and 3 had 2 ligament releases to align the leg to 180°. The iliotibial tract (IT) was the most frequently released structure in all valgus knees (n = 7). As a second release, the posterolateral corner (PLC) was released in 2 patients and the popliteus tendon (POP) in 1 patient.

The IT released as first structure (n = 7) led to a median change in leg axis in extension of 1° towards neutral leg alignment (Figure 5). There was hardly any effect on femoral rotation in flexion: 0.2° endorotation (Figure 6). The PLC released as second structure had no additional effect on the leg axis in extension, and almost no effect on femoral rotation in flexion: 0.15° exorotation. On the other hand, when the POP was released after the IT, this led to an additional change in leg axis in extension of 3° towards neutral leg alignment. The additional change in femoral rotation in flexion was 2.7° endorotation.

**Figure 5.**
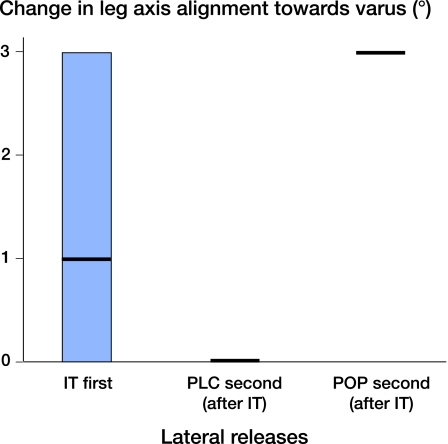
Box plot of change in leg axis after IT release (n = 7) and subsequent PLC (n = 2) or POP release (n = 1) The median is shown as a horizontal line across each box. The vertical lines represent the minimum and maximum values.

**Figure 6.**
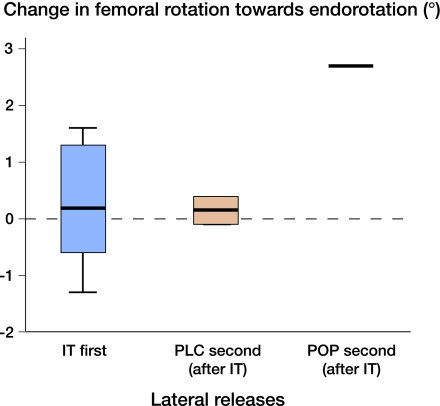
Box plot of change in femoral rotation after IT release (n = 7) and subsequent PLC (n = 2) or POP release (n = 1). The median is shown as a horizontal line across each box. The vertical lines represent the minimum and maximum values.

## Discussion

We have not found any studies in which a balanced gap technique is used during PCL-retaining TKA to describe the effects of ligament releases on leg axis in extension and on femoral rotation in flexion. Our results show the effectiveness of releasing the tightest soft tissue structure first in a clinical TKA situation using the balanced gap technique. In all patients, whether they had a varus or valgus deformity, a neutral leg alignment was achieved. In more than half of the patients, 1 or more ligament releases were necessary. There was no statistically significant difference in the alignment-correcting effect of a release depending upon the sequence in which this structure was released.

The PMC and/or the SMCL were always released when a medial release was necessary. This means that these structures were the tightest in extension at that moment, indicating that they are important stabilizers in the arthritic knee in extension. This is in accordance with what has been written in the literature; these structures have their insertions on, or posterior to, the medial epicondyle (Laprade et al. 2007). The alignment correction of a SMCL release as the first structure showed a high variation in extension and flexion: in 1 knee release of the SMCL had 1° of correction in extension whereas in another knee this was 7°. When the SMCL was released as second structure, the variation was much less. This high variation could be explained by a range of natural deformities. Releases were only performed if necessary, and we did not know in advance how many releases were to be performed. Furthermore, because of the clinical character of the study, it is possible that not all SMCL fibers were resected; the surgeon felt which structure was the tightest and released the ligament until loosening was perceived.

Of all the releases that were performed, the effects were the greatest for the extension gap. This was surprising, since it contrasts with previously published studies that have all reported greater effects in flexion than in extension with distraction of the gaps (Matsueda et al. 1999, Krackow and Mihalko. 1999a,b, Mihalko et al. 2003, Luring et al. 2005, 2006b). In our study, the femur rotated externally 1.5° under distraction of the flexion gap when the SMCL was released as first structure and 2° when it was released as second structure. This is considerably less than has been reported in the literature. We believe that with the balanced gap technique, the complete soft tissue surrounding of the knee joint (medially and laterally) is distracted with the tensor. If one structure (the tightest) is released, the first restraining structure will be replaced by another structure that is the tightest at that moment, but still the gaps will be balanced under distraction. Thus, the effect of a single release may be limited with this implantation technique. Furthermore, the PCL, which was retained in all knees, is a medial stabilizer in flexion and may also have prevented further effects in flexion (Whiteside et al. 2000, Whiteside 2002). Although we learned that major medial releases lead to more internal rotation of the femoral component (Heesterbeek et al. 2009), we would not expect that such a small amount of rotation would have a detrimental effect on patellar tracking.

As we have not found any previous clinical release studies with the balanced gap technique, it is difficult to compare our results to any literature. In contrast to most authors who have investigated release sequences, we found that a maximum of 3 releases had to be performed to achieve neutral leg alignment; other studies examined the additional effects of sequential releases of up to 5 or more structures (Matsueda et al. 1999, Kanamiya et al. 2002, Yagishita et al. 2003, Luring et al. 2005). We found that fewer releases were needed to create neutral leg alignment. In almost half of the knees, no releases were required. This is considerably lower than in another clinical publication reporting that only in one quarter of the cases (all varus knees), no releases were needed; those cases had the least varus alignment (Whiteside et al. 2000). This was not the case in our study: although the median leg alignment was different, the knees that did not need ligament releases had a leg alignment in preoperative standing radiographs ranging from 11° valgus to 13° varus; the patients were therefore not those with the least deformed legs.

The effects of releases can be assessed with varus and valgus stress or with distraction of the gaps (Mihalko et al. 2003). Varus and valgus stresses applied to the knee are fundamentally different to distraction of the extension and flexion gap with a tensor. With valgus stress, for example, one stretches the medial side of the knee and one compresses the lateral side at the same time. On the other hand, when the gap is distracted both the medial and lateral sides of the knee are stretched with equal tension when a bi-compartmental tensor is used. As described, the results of releases vary depending on which measurement condition is used (Krackow and Mihalko. 1999a,b). In our opinion, the gap must be distracted in order to investigate the effect of ligament releases on leg alignment correction since releases are performed to correct leg alignment and not primarily to affect varus-valgus stability.

Release of the PMC and SMCL resulted in similar amounts of correction, independently of the release sequence. Although we cannot make a fair comparison with the literature, cadaver studies have shown that the sequence in which the releases are performed influences the amount of correction of a specific structure (Warren et al. 1974, Nielsen et al. 1984a,b). In contrast to our study, however, these studies were on cadaver knees and with stress applied. One other study did measure leg alignment with a navigation system under gap distraction with a tensor: an effect of 2.7° in extension and 3° in flexion after PMC release was found (Luring et al. 2006b). However, the semimembranosus was also released, which could explain the greater effect; in addition, the study had been performed on cadaver knees without OA. SMCL release resulted in 9° in extension and 11° in flexion, which we could confirm. In both our study and that by Luring et al. (2006b), the PCL—an important medial stabilizer—was intact.

In all lateral releases, the IT was released. In accordance with the literature, this structure is a stabilizer in extension (Whiteside 1999, Peters et al. 2001). Also, the amount of correction was similar to that in a cadaver experiment with gap distraction (Krackow and Mihalko 1999b). Release of the popliteus tendon should not have had an effect in extension (Whiteside 2002), but besides 2.7° in flexion we also found 3° of correction in extension (n = 1).

We released osteophytes first. From previous work, we know that removal of osteophytes influences leg alignment (Yagishita et al. 2003). We did not record the correcting effect of removal of osteophytes, but aimed at describing the effect of ligament releases alone. Because osteophytes should be removed in any case, it would be better to do it first rather than afterwards—which would interfere with the leg alignment just achieved.

The knee joint approach itself can be regarded as a first release, with some effect on leg axis. However, with the current experimental set-up it was not possible to measure this effect because the navigation reference frames can only be put in place after the approach of the joint. Thus, the first measured leg alignment before any release has been performed must not be regarded as the real starting point. The results could, in theory, be different for minimally invasive approaches. However, in our opinion our research questions could be answered using the clinical set-up of this study with patients undergoing TKA. We determined leg alignment in extension and flexion in real time with a navigation system, and could therefore assess the effect of each ligament release step accurately. Thus, we believe that the results of our study can be applied directly to the clinical situation when the balanced gap technique is used in performing a PCL-retaining TKA. Femoral component rotation is not affected when a bone-oriented approach is used. Even so, our findings may be applicable by estimating the change of the gaps in extension and flexion after the desired releases.

## References

[CIT0001] Berger A, Rubash E, Seel J, Thompson H, Crossett S (1993). Determining the rotational alignment of the femoral component in total knee arthroplasty using the epicondylar axis. Clin Orthop.

[CIT0002] Clarke HD, Fuchs R, Scuderi GR, Scott WN, Insall JN (2005). Clinical results in valgus total knee arthroplasty with the ”pie crust” technique of lateral soft tissue releases. J Arthroplasty.

[CIT0003] Fehring TK (2000). Rotational malalignment of the femoral component in total knee arthroplasty. Clin Orthop.

[CIT0004] Fehring TK, Odum S, Griffin WL, Mason JB, Nadaud M (2001). Early failures in total knee arthroplasty. Clin Orthop Relat Res.

[CIT0005] Heesterbeek PJ, Jacobs WC, Wymenga AB (2009). Effects of the balanced gap technique on femoral component rotation in TKA. Clin Orthop.

[CIT0006] Insall J, Ranawat CS, Scott WN, Walker P (1976). Total condylar knee replacment: preliminary report. Clin Orthop.

[CIT0007] Insall JN, Binazzi R, Soudry M, Mestriner LA (1985). Total knee arthroplasty. Clin Orthop.

[CIT0008] Kanamiya T, Whiteside LA, Nakamura T, Mihalko WM, Steiger J, Naito M (2002). Ranawat Award paper. Effect of selective lateral ligament release on stability in knee arthroplasty. Clin Orthop.

[CIT0009] Katz MA, Beck TD, Silber JS, Seldes RM, Lotke PA (2001). Determining femoral rotational alignment in total knee arthroplasty: reliability of techniques. J Arthroplasty.

[CIT0010] Krackow KA, Mihalko WM (1999a). The effect of medial release on flexion and extension gaps in cadaveric knees: implications for soft-tissue balancing in total knee arthroplasty. Am J Knee Surg.

[CIT0011] Krackow KA, Mihalko WM (1999b). Flexion-extension joint gap changes after lateral structure release for valgus deformity correction in total knee arthroplasty: a cadaveric study. J Arthroplasty.

[CIT0012] Laprade RF, Engebretsen AH, Ly TV, Johansen S, Wentorf FA, Engebretsen L (2007). The anatomy of the medial part of the knee. J Bone Joint Surg Am.

[CIT0013] Luring C, Hufner T, Perlick L, Bathis H, Krettek C, Grifka J (2005). Soft tissue management in knees with varus deformity Computer-assisted sequential medial ligament release. Orthopade.

[CIT0014] Luring C, Bathis H, Hufner T, Grauvogel C, Perlick L, Grifka J (2006a). Gap configuration and anteroposterior leg axis after sequential medial ligament release in rotating-platform total knee arthroplasty. Acta Orthop.

[CIT0015] Luring C, Hufner T, Perlick L, Bathis H, Krettek C, Grifka J (2006b). The effectiveness of sequential medial soft tissue release on coronal alignment in total knee arthroplasty: using a computer navigation model. J Arthroplasty.

[CIT0016] Matsueda M, Gengerke TR, Murphy M, Lew WD, Gustilo RB (1999). Soft tissue release in total knee arthroplasty. Cadaver study using knees without deformities. Clin Orthop.

[CIT0017] Mihalko WM, Whiteside LA, Krackow KA (2003). Comparison of ligament-balancing techniques during total knee arthroplasty. J Bone Joint Surg Am (Suppl 4).

[CIT0018] Nielsen S, Kromann-Andersen C, Rasmussen O, Andersen K (1984a). Instability of cadaver knees after transection of capsule and ligaments. Acta Orthop Scand.

[CIT0019] Nielsen S, Rasmussen O, Ovesen J, Andersen K (1984b). Rotatory instability of cadaver knees after transection of collateral ligaments and capsule. Arch Orthop Trauma Surg.

[CIT0020] Olcott CW, Scott RD (2000). A comparison of 4 intraoperative methods to determine femoral component rotation during total knee arthroplasty. J Arthroplasty.

[CIT0021] Peters CL, Mohr RA, Bachus KN (2001). Primary total knee arthroplasty in the valgus knee: creating a balanced soft tissue envelope. J Arthroplasty.

[CIT0022] Poilvache PL, Insall JN, Scuderi GR, Font-Rodriguez DE (1996). Rotational landmarks and sizing of the distal femur in total knee arthroplasty. Clin Orthop.

[CIT0023] Saeki K, Mihalko WM, Patel V, Conway J, Naito M, Thrum H, Vandenneuker H, Whiteside LA (2001). Stability after medial collateral ligament release in total knee arthroplasty. Clin Orthop.

[CIT0024] Warren A, Marshall L, Girgis F (1974). The prime static stabilizer of the medical side of the knee. J Bone Joint Surg (Am).

[CIT0025] Whiteside LA (1999). Selective ligament release in total knee arthroplasty of the knee in valgus. Clin Orthop.

[CIT0026] Whiteside LA (2002). Soft tissue balancing: the knee. J Arthroplasty.

[CIT0027] Whiteside LA, Arima J (1995). The anteroposterior axis for femoral rotational alignment in valgus total knee arthroplasty. Clin Orthop.

[CIT0028] Whiteside LA, Saeki K, Mihalko WM (2000). Functional medial ligament balancing in total knee arthroplasty. Clin Orthop.

[CIT0029] Yagishita K, Muneta T, Ikeda H (2003). Step-by-step measurements of soft tissue balancing during total knee arthroplasty for patients with varus knees. J Arthroplasty.

